# Metabolomics and proteomics reveal drought-stress responses of leaf tissues from spring-wheat

**DOI:** 10.1038/s41598-018-24012-y

**Published:** 2018-04-09

**Authors:** Anna Michaletti, Mohammad Reza Naghavi, Mahmoud Toorchi, Lello Zolla, Sara Rinalducci

**Affiliations:** 1 Department of Ecological and Biological Sciences (DEB), University of Tuscia, Viterbo, Italy; 2 Department of Agriculture, Payame Noor University, Tehran, Iran; 3 Department of Biotechnology and Plant Breeding, University of Tabriz, Tabriz, Iran; 4 Department of Science and Technology for Agriculture, Forestry, Nature and Energy (DAFNE), University of Tuscia, Viterbo, Italy

## Abstract

To reveal the integrative biochemical networks of wheat leaves in response to water deficient conditions, proteomics and metabolomics were applied to two spring-wheat cultivars (*Bahar*, drought-susceptible; *Kavir*, drought-tolerant). Drought stress induced detrimental effects on Bahar leaf proteome, resulting in a severe decrease of total protein content, with impairments mainly in photosynthetic proteins and in enzymes involved in sugar and nitrogen metabolism, as well as in the capacity of detoxifying harmful molecules. On the contrary, only minor perturbations were observed at the protein level in Kavir stressed leaves. Metabolome analysis indicated amino acids, organic acids, and sugars as the main metabolites changed in abundance upon water deficiency. In particular, Bahar *cv* showed increased levels in proline, methionine, arginine, lysine, aromatic and branched chain amino acids. Tryptophan accumulation via shikimate pathway seems to sustain auxin production (indoleacrylic acid), whereas glutamate reduction is reasonably linked to polyamine (spermine) synthesis. Kavir metabolome was affected by drought stress to a less extent with only two pathways significantly changed, one of them being purine metabolism. These results comprehensively provide a framework for better understanding the mechanisms that govern plant cell response to drought stress, with insights into molecules that can be used for crop improvement projects.

## Introduction

Drought is one of the most important environmental factors that limit worldwide plant performance, growth and productivity^1^. On the other hand, as the population of the world increases exponentially and the adverse alterations in climate that impact crop productivity become more intense, the agriculture sector is facing a major challenge of ensuring a sufficient food supply to the masses. Consequently, breeding programs for plant varieties adapted to various environmental stresses increased and researchers are interested to molecularly dissect complex traits conferring stress tolerance or susceptibility. Drought stress induces a number of profound changes at the morphological, physiological and biochemical level in all plant organs^2^, basically disturbing the relationship between sink and source plant organs. Plant stress response represents a complex and highly dynamic process aimed at establishing a novel homeostasis under unfavorable growth conditions. Specifically, drought-responsive mechanisms include hormone induction, signaling of kinase cascade, gene expression regulation, reactive oxygen species scavenging, osmolyte synthesis, cell structure modulation, activation of ion channels, carbohydrate and energy metabolism, nitrogen assimilation and amino acid metabolism, as well as fatty acid metabolism^3,4^. This active process involves genes, proteins and small molecules (metabolites), but the latter play a crucial role as being directly involved in plant cell structure and metabolism by shaping the final phenotype^5^. Thus, a precise and simultaneous analysis of the proteome and metabolome in drought tolerant and susceptible plant cultivars is essential for understanding the fundamentals of stress adaptation physiology and biochemistry. It is commonly accepted that the high-throughput “omics” techniques have enabled researchers to study plant responses to environmental stresses in a more holistic view. More specifically, it is the integration of such comprehensive approaches (the so called systems biology) that is allowing the elucidation of the composite regulatory network activated by plants in response to external factors including abiotic stresses^5–7^.

The major socioeconomically crops affected by water-limiting conditions are grown in temperate climate and include cereals, *i*.*e*. common (*Triticum aestivum*) and durum (*Triticum durum*) wheat, barley (*Hordeum vulgare*), maize (*Zea mays*) and rice (*Oryza sativa*). Proteomics has been extensively applied to study drought responsive pathways in leaves of such plants^8–22^, whereas investigations on metabolic adjustments are still limited^22–28^.

Our study dealt with common wheat that remains the most grown crop worldwide with the third highest total production of *ca*. 729 million metric tons (http://www.fao.org/faostat/en/#data/QC). In some wheat producer countries, drought stress represents the most relevant agronomic problem, as they present wide zones lacking a satisfactory and constant amount of rainfalls. Iran is an arid and semi-arid country located in the desert belt of northern hemisphere, however it is the eleventh most producer and the seventh most consumer country of wheat in the world. Here and in similar regions, scientists are expecting to produce superior wheat lines able to tolerate water deficit stress. Our work is inserted in this context and focused on the comparative analysis of drought-related protein and metabolite abundance between two Iranian native wheat cultivars with different degree of drought tolerance. In particular, according to trait classifications reported by Naghavi *et al*.^29^, the spring-habit pure lines that we used are among the most drought-tolerant (Kavir) and -susceptible (Bahar) wheat varieties developed by CIMMYT (International Maize and Wheat Improvement Center) for dry and temperate regions in the world. Alterations in the proteome were investigated by classical two-dimensional gel electrophoresis coupled with mass spectrometry identification of differentially modulated spots, whereas metabolite changes were studied through direct LC-MS-based untargeted metabolomics methods. Under the stress conditions imposed in our study (7-days of water deficit), findings underlined a state of particular sufferance in the sensitive Bahar cultivar that was mainly explicated with: (i) damages to photosynthesis, (ii) alterations in carbon partioning and nitrogen assimilation, (iii) impairment of detoxification activities. However, specific defense attempts, such as an increased production of pipecolic acid, spermine and tryptophan-derived auxins were also highlighted. On the contrary, the drought-tolerant plants showed a remarkable stability both at the protein and metabolic level.

## Results

### Morpho-physiological traits

**Figure 1:**
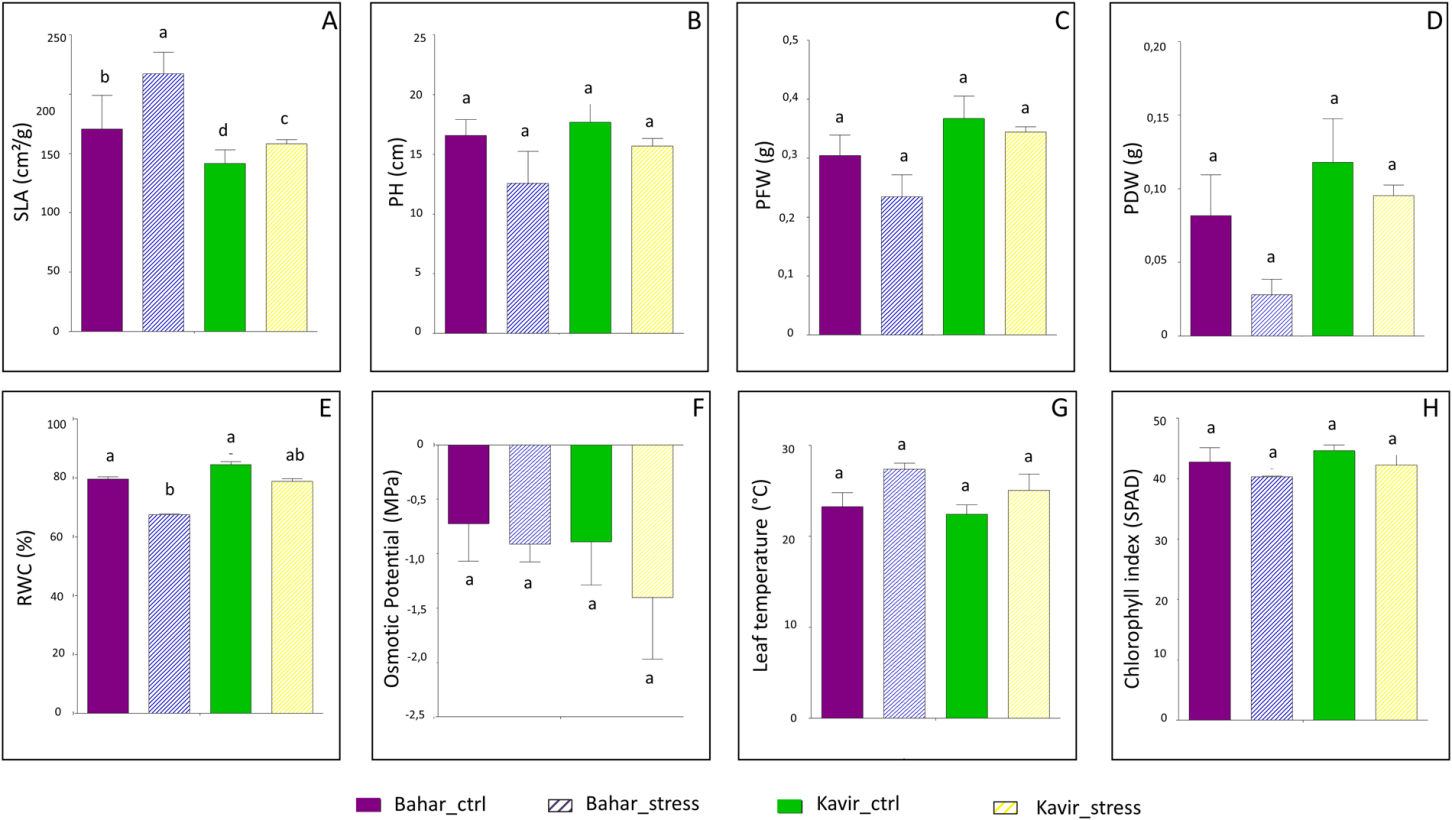
Mean comparison of morphological and physiological traits. Data are means of three replicates ± SD. The different letters indicate significant difference (p ≤ 0.05) by Duncan’s test. SLA, specific leaf area (**A**); PH, plant height (**B**); PFW, plant fresh weight (**C**); PDW, plant dry weight (**D**); RWC, relative water content (**E**); Osmotic potential (**F**); Leaf temperature (**G**); Chlorophyll index (**H**). Ctr, control.

For eight morpho-physiological traits (specific leaf area, plant height, plant fresh and dry weight, relative water content, osmotic potential, leaf temperature, chlorophyll index), analysis of variance was assessed at the stage of seedlings in Bahar (susceptible) and Kavir (tolerant) wheat varieties. The two cultivars showed statistically significant differences for all the evaluated traits between normal irrigation and drought stress conditions (p < 0.0001). Genotype and treatment variability was assessed by coefficient of variation measurements as reported in Supplementary Table S1. Figure 1 shows a comparison between means of all traits. The results were based on three replicates and data were subjected to ANOVA (p < 0.05) followed by post-hoc Duncan’s test. Under well-watered condition, the two cultivars did not show significant difference except for specific leaf area (SLA) in which the susceptible cultivar exhibited higher mean compared to the tolerant cultivar. Under drought stress condition the two cultivars indicated a decrease in all traits except for SLA and leaf temperature (Fig. 1). Also, under water deficiency the decrease in PFW (plant fresh weight), PDW (plant dry weight) and RWC (relative water content) is considerably more in the susceptible cultivar (Bahar) than in the tolerant one (Fig. 1).

### Differential proteomics analysis

**Figure 2:**
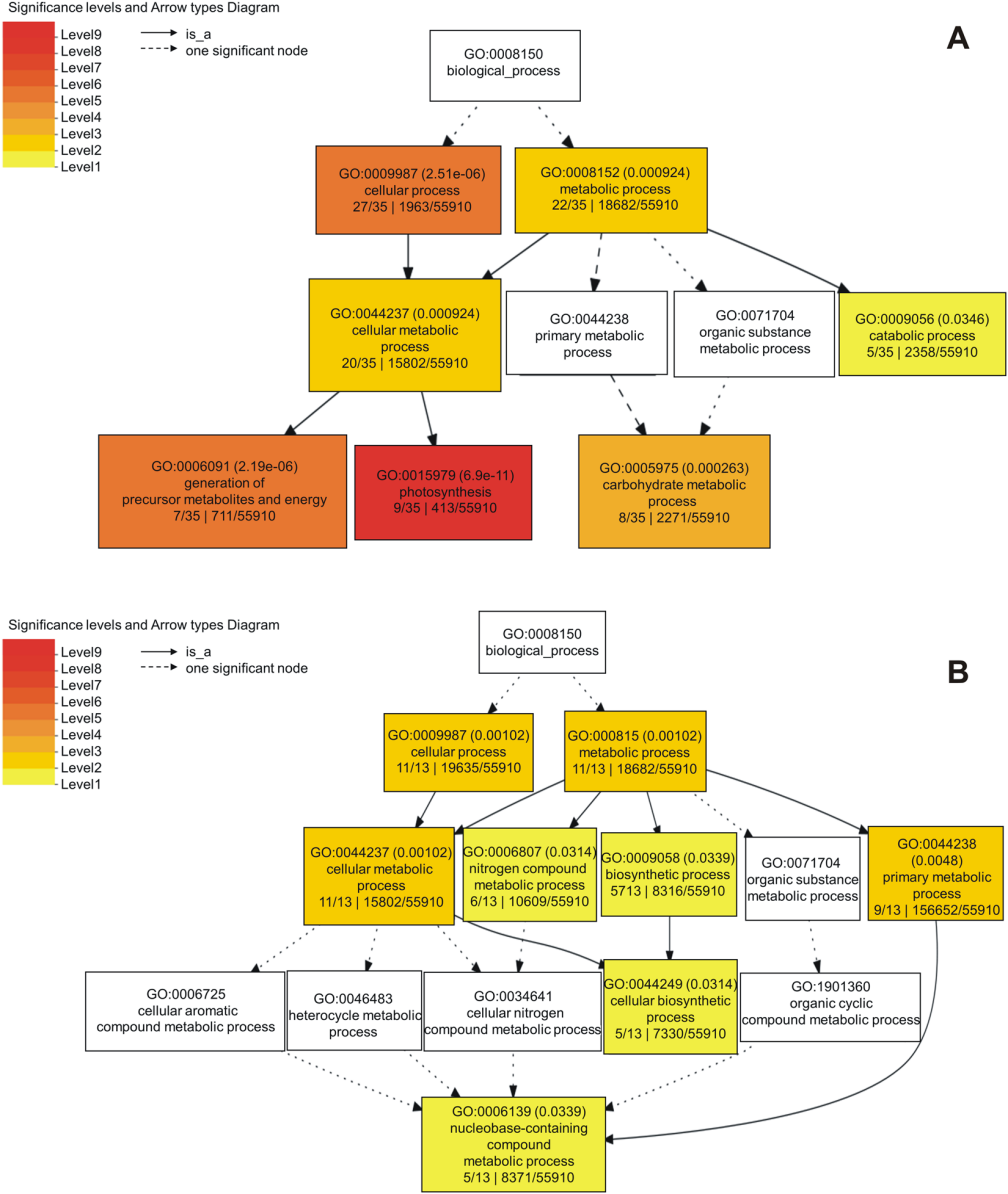
Gene Ontology (GO) analysis of drought stress modulated proteins. Classification was performed in Bahar (panel A) and Kavir (panel B) cultivars using agriGO. Boxes represent GO terms labelled by GO number, term definition and statistical information (adjusted p-value in parenthesis). Significant terms (FDR < 0.05) are coloured. The degree of colour saturation of a box is positively correlated to the enrichment level of the term.

Drought stress related proteins were investigated by 2D-PAGE analysis. Two comparisons were conducted: (i) normal irrigation versus water-deficit condition in drought-stress sensitive cultivar (Bahar); (ii) well-watered versus drought-stress condition in tolerant wheat cultivar (Kavir). Overall, a decrease in the total protein content was observed after drought stress. This feature is generally considered a senescence parameter (impairment of protein synthesis and increase of protein degradation). Our comparative analysis revealed a total of 85 differentially abundant protein spots (p < 0.05; fold change ≥1.5) in the Bahar comparison, whereas 20 protein spots exhibited significant changes in abundance in Kavir (Supplementary Fig. S1). Variable spots (indicated with numbers in Supplementary Fig. S1) were excised from the gels, digested by trypsin and peptide mixtures were then analyzed by LC-ESI-MS/MS for protein identification. The positively identified proteins are listed in Table 1 (relative to Bahar cultivar) and 2 (relative to Kavir cultivar), together with the protein spot number and the identification parameters. As regards to Bahar comparison, of the 81 identified protein spots (success rate of 95%), 72 were unique proteins. On the contrary, protein LC-MS/MS analysis in Kavir cultivar led to the detection of 8 unique proteins among those positively identified that were 17. All the differentially expressed proteins detected after 7 days of water withholding were found to be down-regulated, both in susceptible and tolerant common wheat variety. In order to identify the relevant drought-altered pathways, we performed an enrichment analysis for biological processes by using the SEA (Singular Enrichment Analysis) tool in agriGO database (Fig. 2A,B). GO classification results are displayed as a hierarchical tree, leading to some redundancy. Therefore, all significantly enriched GO terms (FDR < 0.05) from agriGO analysis were submitted to the REVIGO program in order to reduce the redundancy and allow visualization of the most informative common ancestor nodes (Supplementary Fig. S2). Among significantly altered biological processes, of particular note are photosynthesis, carbohydrate metabolic process, and nitrogen compound metabolic process. However, due to lack of GO annotation availability for some entries, the agriGO analysis lost some interesting proteins in terms of drought stress response. Representative proteins for each of these categories will be discussed.

**Table 1:** List of differentially abundant proteins identified by LC-MS/MS in Bahar cultivar.

Spot No.^a^	Accession number	Protein name	Mascot score	Match^b^	Unique Sequences^b^	Seq. Coverage	Theoretical/Experimental Mr (kDa)	Theoretical/Experimental pI	Taxonomy	Fold change^c^	One-way ANOVA (p-value)
351	EMS68298.1	RuBisCO large subunit-binding protein subunit beta, chloroplastic	1680	43(43)	21(21)	31%	83.354/60	7.79/5.3	Triticum urartu	2.6	0.019
354	EMS68298.1	RuBisCO large subunit-binding protein subunit beta, chloroplastic	1672	46(46)	21(21)	32%	83.354/60	7.79/5.25	Triticum urartu	2.9	0.005
465	AAP92166.1	Ribulose-1,5-bisphosphate carboxylase/oxygenase large subunit (chloroplast)	1217	34(34)	17(17)	32%	53.442/45	6.22/6.55	Triticum aestivum	1.7	0.042
466	NP_114267.1	Ribulose-1,5-bisphosphate carboxylase/oxygenase large subunit (chloroplast)	926	29(29)	14(14)	29%	53.445/45	6.22/6.35	Triticum aestivum	2.8	0.013
495	EMS49604.1	Glyceraldehyde-3-phosphate dehydrogenase B, chloroplastic	498	11(11)	7(7)	18%	47.329/42	6.03/5.2	Triticum urartu	2.4	0.049
623	XP_020184677.1	Fructose-bisphosphate aldolase, chloroplastic-like	1231	52(52)	17(17)	45%	41.837/38	6.08/5.5	Aegilops tauschii subsp. tauschii	3.4	0.012
1150	AAN27974.1	Ribulose-1,5-bisphosphate carboxylase/oxygenase large subunit (chloroplast)	1357	49(49)	19(19)	42%	53.739/45	6.22/6.45	Hordeum comosum	3.6	0.037
547	EMS57012.1	Ribulose bisphosphate carboxylase/oxygenase activase A, chloroplastic	869	35(35)	11(11)	33%	51.235/41	6.90/5.6	Triticum urartu	1.9	0.048
549	EMS57012.1	Ribulose bisphosphate carboxylase/oxygenase activase A, chloroplastic	1306	58(58)	16(16)	45%	51.235/41	6.90/5.45	Triticum urartu	2.0	0.011
552	P26302.1	Phosphoribulokinase, chloroplastic	298	9(9)	4(4)	11%	45.512/41	5.72/5.2	Triticum aestivum	2.0	0.050
557	P26302.1	Phosphoribulokinase, chloroplastic	490	18(18)	7(7)	17%	45.512/39	5.72/5	Triticum aestivum	1.7	0.003
558	P26302.1	Phosphoribulokinase, chloroplastic	673	25(25)	9(9)	21%	45.512/41	5.72/5.1	Triticum aestivum	2.5	0.003
562	EMS57012.1	Bisphosphate carboxylase/oxygenase activase A, chloroplastic	705	19(19)	9(9)	29%	51.235/40	6.90/5.5	Triticum urartu	2.7	0.017
579	P46285.1	Sedoheptulose-1,7-bisphosphatase, chloroplastic	632	28(28)	9(9)	25%	42.547/40	6.04/5	Triticum aestivum	3.3	0.006
581	P46285.1	Sedoheptulose-1,7-bisphosphatase, chloroplastic	2037	73(73)	17(17)	41%	42.547/40	6.4/5	Triticum aestivum	2.9	0.004
587	EMS57012.1	Ribulose bisphosphate carboxylase/oxygenase activase A, chloroplastic	571	13(13)	8(8)	26%	51.235/39	6.90/5.5	Triticum urartu	2.2	0.014
593	CDX48685.1	RuBisCO activase beta, partial	1098	30(30)	14(14)	38%	41.655/38	5.80/6.4	Triticum aestivum	2.0	0.013
607	XP_006662769.1	PREDICTED: fructose-bisphosphate aldolase, chloroplastic	393	13(13)	5(5)	14%	42.208/38	6.38/5.75	Oryza brachyantha	2.2	0.049
613	EMS57012.1	Ribulose bisphosphate carboxylase/oxygenase activase, chloroplastic	774	32(32)	9(9)	24%	51.235/38	6.90/6.5	Triticum urartu	1.5	0.043
619	EMS47455.1	Fructose-bisphosphate aldolase, chloroplastic	632	18(18)	8(8)	25%	42.205/38	5.94/5.3	Triticum urartu	4.1	0.005
655	EMT00761.1	50 S ribosomal protein L4, chloroplastic	239	7(7)	3(3)	11%	31.104/35	5.27/5.4	Aegilops tauschii	2.4	0.047
835	CDX48685.1	RuBisCO activase beta, partial	148	3(3)	2(2)	9%	41.655/28	5.80/5.9	Triticum aestivum	2.1	0.044
988	CAA40669.1	Oxygen-evolving enhancer protein 2, chloroplastic	142	4(4)	2(2)	5%	27.424	8.84	Triticum aestivum	1.6	0.005
1147	P08823.1	RuBisCO large subunit-binding protein subunit alpha, chloroplastic	1944	72(72)	24(24)	51%	57.656/55	4.83/4.9	Triticum aestivum	2.9	0.023
1151	XP_020187838.1	Fructose-1,6-bisphosphatase, chloroplastic	140	5(5)	2(2)	8%	44.785/64.88	5.10/4.9	Aegilops tauschii subsp. tauschii	2.3	0.050
1154	CAC85479.1	Adenosine diphosphate glucose pyrophosphatase	137	3(3)	2(2)	15%	21.972	5.68	Triticum aestivum	2.6	0.004
1157	AAN27974.1	Ribulose-1,5-bisphosphate carboxylase/oxygenase large subunit (chloroplast)	1031	41(41)	14(14)	29%	53.739/45	6.22/6.5	Hordeum comosum	1.6	0.037
1169	EMS57012.1	Ribulose bisphosphate carboxylase large chain	336	14(14)	9(9)	22%	53.739/45	6.22/6.5	Hordeum vulgare	2.8	0.013
1195	EMS57012.1	Ribulose bisphosphate carboxylase large chain	1946	57(57)	22(22)	47%	53.721/45	4.88/5	Secale cereale	2.3	0.027
1214	EMS57012.1	Ribulose bisphosphate carboxylase/oxygenase activase A, chloroplastic	2111	59(59)	19(19)	36%	53.739/45	6.22/6.5	Hordeum vulgare	2.4	0.040
1228	P46285.1	Sedoheptulose-1,7-bisphosphatase, chloroplastic	185	7(7)	3(3)	6%	42.547/40	6.04/5.3	Triticum aestivum	1.6	0.048
1272	EMT15798.1	Putative RuBisCO large subunit-binding protein subunit alpha, chloroplastic	80	1(1)	1(1)	2%	60.954/30	5.00/5.00	Aegilops tauschii	2.8	0.048
398	EMS55427.1	ATP-dependent zinc metalloprotease FTSH 2, chloroplastic	183	3(3)	3(3)	4%	71.987/54	5.7/5.3	Triticum urartu	3.6	0.050
578	EMT19451.1	Carbonic anhydrase, chloroplastic	260	5(5)	4(4)	23%	22.653/41	5.90/5.00	Aegilops tauschii	2.3	0.002
631	XP_020163505.1	Photosystem II stability/assembly factor HCF136, chloroplastic	1008	29(29)	12(12)	34%	42.03/37	6.47/5.1	Agrostis tenerrima	2.2	0.017
704	ABQ52657.1	Oxygen-evolving enhancer protein 1, chloroplastic	459	10(10)	6(6)	25%	34.635/35	5.75/5.00	Triticum urartu	4.3	9.48 × 10^−4^
709	ABQ52657.1	Oxygen-evolving enhancer protein 1, chloroplastic	538	13(13)	7(7)	28%	34.635/35	5.75/4.9	Triticum urartu	3.8	0.029
710	XP_020186778.1	Oxygen-evolving enhancer protein 1, chloroplastic	791	41(41)	11(11)	52%	34.635/35	5.75/5.2	Triticum urartu	3.4	0.002
714	ABQ52657.1	Oxygen-evolving enhancer protein 1, chloroplastic	762	25(25)	11(11)	36%	34.635/35	5.75/5.00	Triticum urartu	3.6	0.024
716	XP_020186778.1	Oxygen-evolving enhancer protein 1, chloroplastic	905	40(40)	13(13)	53%	34.635/35	5.75/5.1	Triticum urartu	2.9	0.007
825	EMS46089.1	Thylakoid lumenal 29 kDa protein, chloroplastic	495	17(17)	6(6)	23%	38.454	7.59	Triticum urartu	3.0	0.006
876	AFS34654.1	Chloroplast chlorophyll a-b binding protein, partial	400	18(18)	5(5)	34%	20.709/27	6.34/5.5	Leymus secalinus	2.1	0.007
868	EMT19451.1	Carbonic anhydrase, chloroplastic	129	2(2)	2(2)	13%	22653/26	5.97/5.6	Aegilops tauschii	2.6	0.007
872	CAC94002.1	Glutathione transferase	152	4(4)	2(2)	9%	25.108/28	6.35/6.5	Triticum aestivum	2.8	0.031
875	AFS34654.1	Chloroplast chlorophyll a-b binding protein, partial	272	10(10)	4(4)	33%	20.709/29	634/5.7	Leymus secalinus	3.5	0.004
928	ACO06083.1	Chlorophyll a-b binding protein	130	4(4)	2(2)	9%	26.732/23	5.42/5.3	Triticum aestivum	2.7	0.042
929	Q00434.1	Oxygen-evolving enhancer protein 2, chloroplastic	225	5(5)	3(3)	10%	27.424	8.84/	Triticum aestivum	2.5	0.039
1148	Q00434.1	Oxygen-evolving enhancer protein 2, chloroplastic	720	34(34)	10(10)	37%	27.424	5.91/6	Triticum tauschii	2.5	0.021
1218	AAL75812.1	Temperature stress-induced lipocalin	225	4(4)	3(3)	18%	21.809	5.5/5	Triticum aestivum	2.2	0.050
1161	ABQ52657.1	Chloroplast oxygen-evolving enhancer protein 1	166	4(4)	2(2)	8%	34.719/24	6.08/6.3	Leymus chinensis	2.5	0.047
1162	EMS54912.1	PsbP domain-containing protein, chloroplastic	211	4(4)	2(2)	11%	27.741/24	5.48/6.4	Triticum urartu	3.0	0.034
1173	XP_020201330.1	Cytochrome b6-f complex iron-sulfur subunit, chloroplastic	252	6(6)	4(4)	15%	24.110/22	8.47/6.25	Triticum aestivum	3.6	0.025
1232	EMS59167.1	Peptidyl-prolyl cis-trans isomerase CYP38, chloroplastic	330	8(8)	5(5)	13%	46.100/40	4.82/4.7	Triticum urartu	1.8	0.041
1313	EMS59167.1	Peptidyl-prolyl cis-trans isomerase CYP38, chloroplastic	347	8(8)	5(5)	11%	46.100/40	4.82/4.7	Triticum urartu	5.1	0.017
491	ACT22496.1	plastid glutamine synthetase 2	217	5(5)	3(3)	12%	47.002/43	5.75/4.8	Triticum aestivum	2.6	0.032
498	P13564.2	Glutamine synthetase leaf isozyme, chloroplastic	123	2(2)	2(2)	8%	47.016/41	5.11/4.9	Hordeum vulgare	2.3	0.005
499	ACT22500.1	Plastid glutamine synthetase 2	838	25(25)	10(10)	36%	46.986/41	5.75/5.1	Triticum aestivum	2.6	4.54 × 10^−4^
507	P13564.2	Glutamine synthetase leaf isozyme, chloroplastic	72	3(3)	1(1)	3%	47.406/41	5.11/5.00	Hordeum vulgare	2.7	0.002
601	P13564.2	Glutamine synthetase leaf isozyme, chloroplastic	412	13(13)	6(6)	19%	47.406/36	5.11/5.00	Hordeum vulgare	2.4	0.031
604	P13564.2	Full = Glutamine synthetase leaf isozyme, chloroplastic	431	12(12)	6(6)	19%	47.406/38	5.11/4.9	Hordeum vulgare	3.6	2.40 × 10^−4^
784	XP_020192093.1	Salt stress root protein RS1	116	3(3)	2(2)	12%	22.168/31	4.82/4.8	Aegilops tauschii subsp. tauschii	2.0	0.044
1152	P13564.2	Glutamine synthetase leaf isozyme, chloroplastic	207	6(6)	3(3)	12%	47.406/42	5.11/4.9	Hordeum vulgare	2.7	0.013
395	NP_114266.1	ATP synthase CF1 beta subunit	1232	44(44)	15(15)	38%	53.881/57	5.06/5.25	Triticum aestivum	3.9	3.25 × 10^−4^
396	NP_114266.1	ATP synthase CF1 beta subunit	1714	85(85)	21(21)	57%	53.881/56	5.06/5.2	Triticum aestivum	3.1	0.007
397	NP_114266.1	ATP synthase CF1 beta subunit	1609	66(66)	19(19)	53%	53.881/56	5.06/5.1	Triticum aestivum	2.6	0.012
402	CAA52636.1	ATP synthase beta subunit	844	27(27)	12(12)	31%	59.33/50	5.56/5.3	Triticum aestivum	2.8	0.037
1226	XP_020171603.1	Probable ATP synthase 24 kDa subunit, mitochondrial	601	21(21)	8(8)	39%	27.051/29	7.71/5.6	Aegilops tauschii subsp. tauschii	2.4	0.039
722	XP_020167925.1	Soluble inorganic pyrophosphatase 6, chloroplastic	327	6(6)	5(5)	17%	31.825/32	5.6/5	Aegilops tauschii subsp. tauschii	2.6	0.043
965	EMS51159.1	Germin-like protein 8–14	56	1(1)	1(1)	4%	22.096/24	5.37/6	Triticum urartu	2.9	0.012
840	P46226.3	Full = Triosephosphate isomerase, Cytosolic	79	2(2)	2(2)	10%	27.138/28	5.24/5.8	Secale cereale	3.0	0.028
635	EMT17715.1	Malate dehydrogenase 1, Mitochondrial	390	17(17)	5(5)	14%	34.931/36	5.26/6.3	Aegilops tauschii	1.9	0.045
1179	EMT17715.1	Malate dehydrogenase 1, mitochondrial	476	15(15)	6(6)	21%	34.931/36	5.26/6.5	Aegilops tauschii	2.9	0.047
911	EMS52570.1	Putative glutathione S-transferase GSTU1	61	1(1)	1(1)	5%	25.112/25	6.36/6.00	Triticum urartu	1.9	0.028
667	CAB50787.2	Putative glyoxalase I, partial	249	6(6)	4(4)	14%	31.830/36	5.39/5.4	Triticum aestivum	1.9	0.050
1159	EMS51416.1	Heat shock 70 kDa protein, mitochondrial	638	13(13)	9(9)	16%	76.357/60	6.16/5.4	Triticum urartu	1.6	0.003
681	EMT31279.1	Alpha-soluble NSF attachment protein	623	14(14)	7(7)	28%	35.156/35	4.96/4.9	Aegilops tauschii	1.8	0.024
693	AGH18694.1	Tetratricopeptide repeat containing protein	381	8(8)	6(6)	19%	36.528/33	7.49/5.35	Triticum monococcum	2.3	0.031
852	EMS51246.1	Proteasome subunit alpha type-2	235	6(6)	3(3)	6%	41.129/30	6.05/5.3	Triticum urartu	2.0	0.007
745	EMT30761.1	Protein grpE	318	8(8)	4(4)	14%	33.986/33	5.5/5.2	Aegilops tauschii	2.7	0.005
770	AAR26488.1	Harpin binding protein-1	465	12(12)	6(6)	29%	29.079/31	7.77/5	Triticum aestivum	1.9	0.029
802	CBI30539.3	Unnamed protein product, partial	116	2(2)	2(2)	5%	27.682/31	5.85/5.6	Vitis vinifera	1.8	0.001

^a^Spot numbers refer to Supplementary Figure S1; ^b^Values between parentheses indicate statistically significant peptides (p < 0.05); ^c^Fold of protein variation refers to stressed *vs* control. All spots resulted to be down-modulated.

### Differential metabolomics analysis

**Figure 3:**
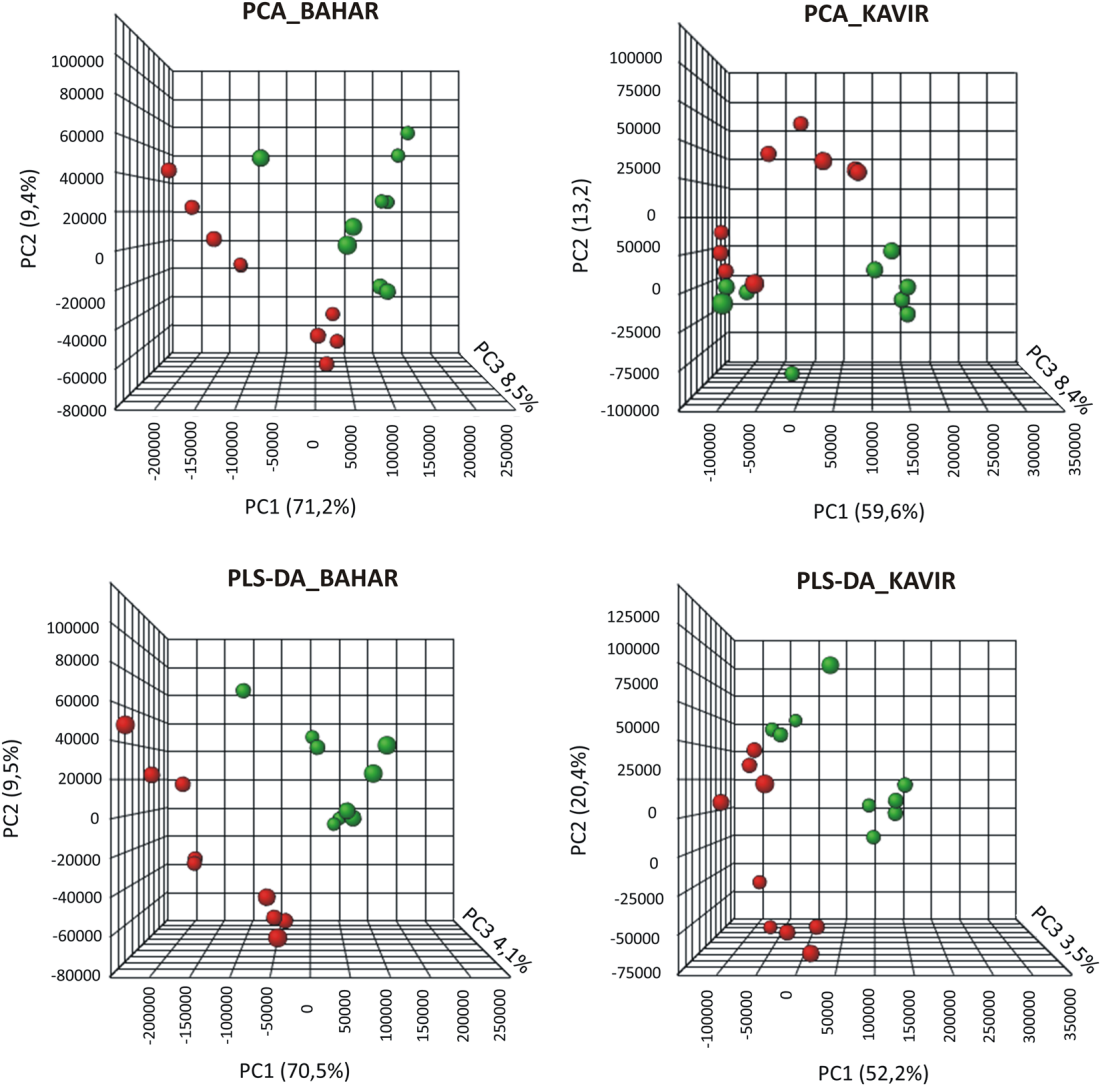
Multivariate statistical analysis of metabolomics data from Bahar and Kavir wheat leaves. Three dimensional Principal Component Analysis (PCA) and Partial Least Square Discriminant Analysis (PLS-DA) score plots are shown in the upper and lower panels, respectively. Control sample groups are in red; drought-stressed sample groups are in green.

**Figure 4:**
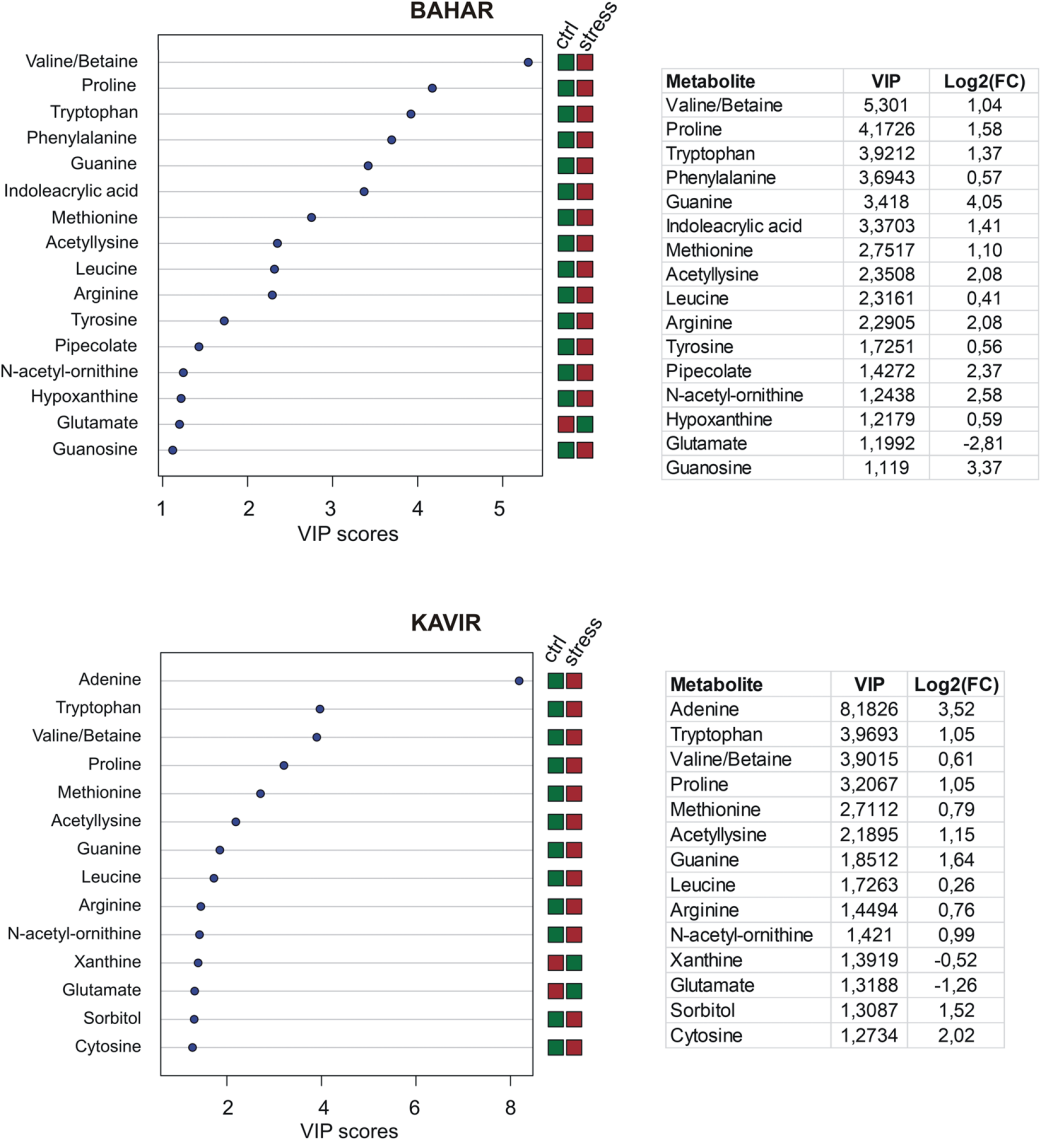
Important features identified by PLS-DA through MetaboAnalyst web-based platform. Colored boxes indicate the relative concentrations of the corresponding metabolite in each group under current study (red, up-regulation; green, down-regulation). Tables on the right report the VIP (Variable Importance in the Projection) values for Component 1 and the fold changes in the concentrations of each metabolite. Fold changes were calculated using the formula log2(drought stressed/control). Ctr, control.

**Figure 5:**
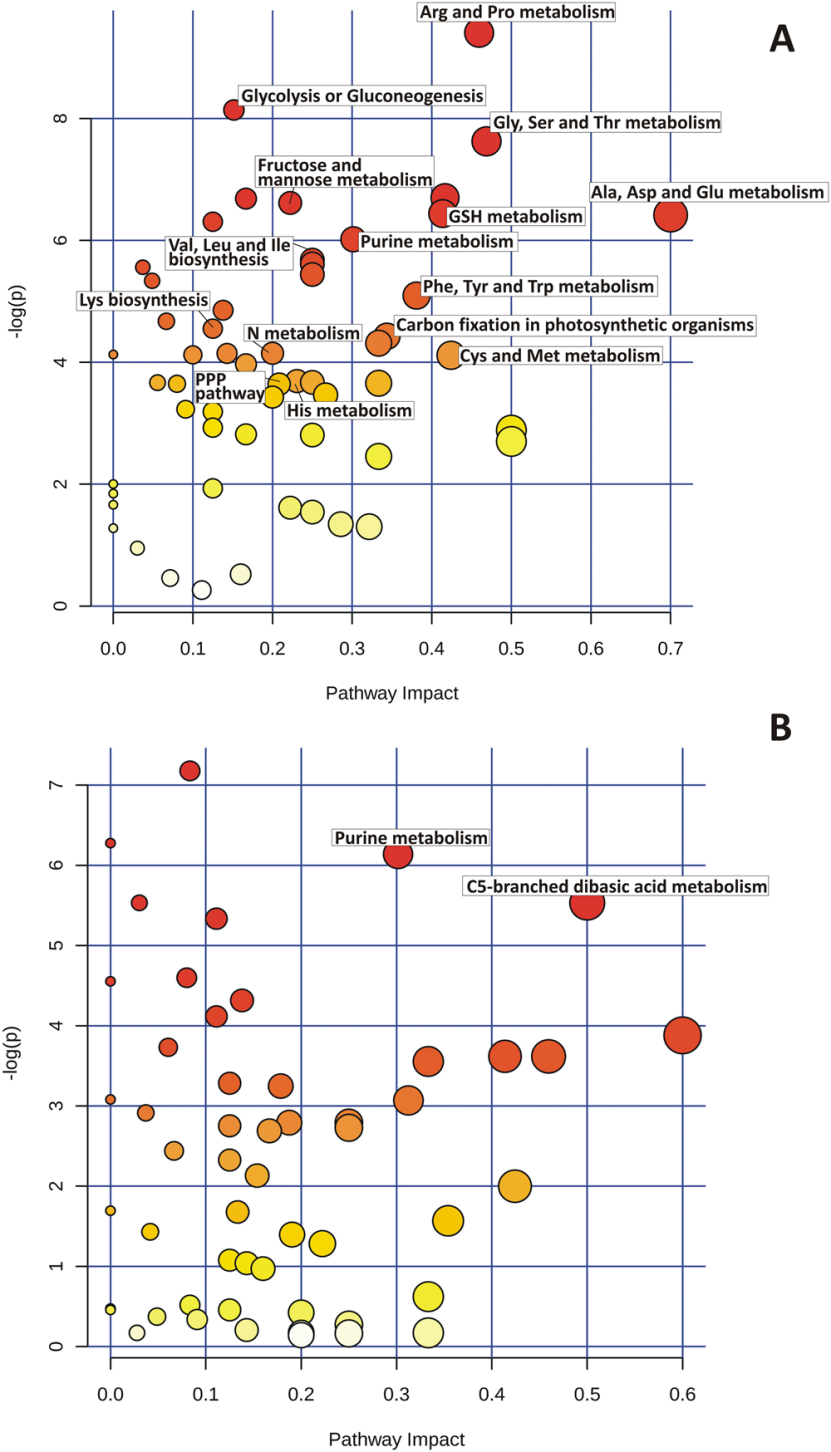
Metabolomic Pathway Analysis (MetPA) as generated by MetaboAnalyst software package. Panels A and B show results for Bahar and Kavir metabolite data sets, respectively. All the matched pathways are displayed as circles. The color of each circle is based on p-values (darker colors indicate more significant changes of metabolites in the corresponding pathway), whereas the size of the circle corresponds to the pathway impact score. The most impacted pathways having high statistical significance scores are annotated.

**Figure 6:**
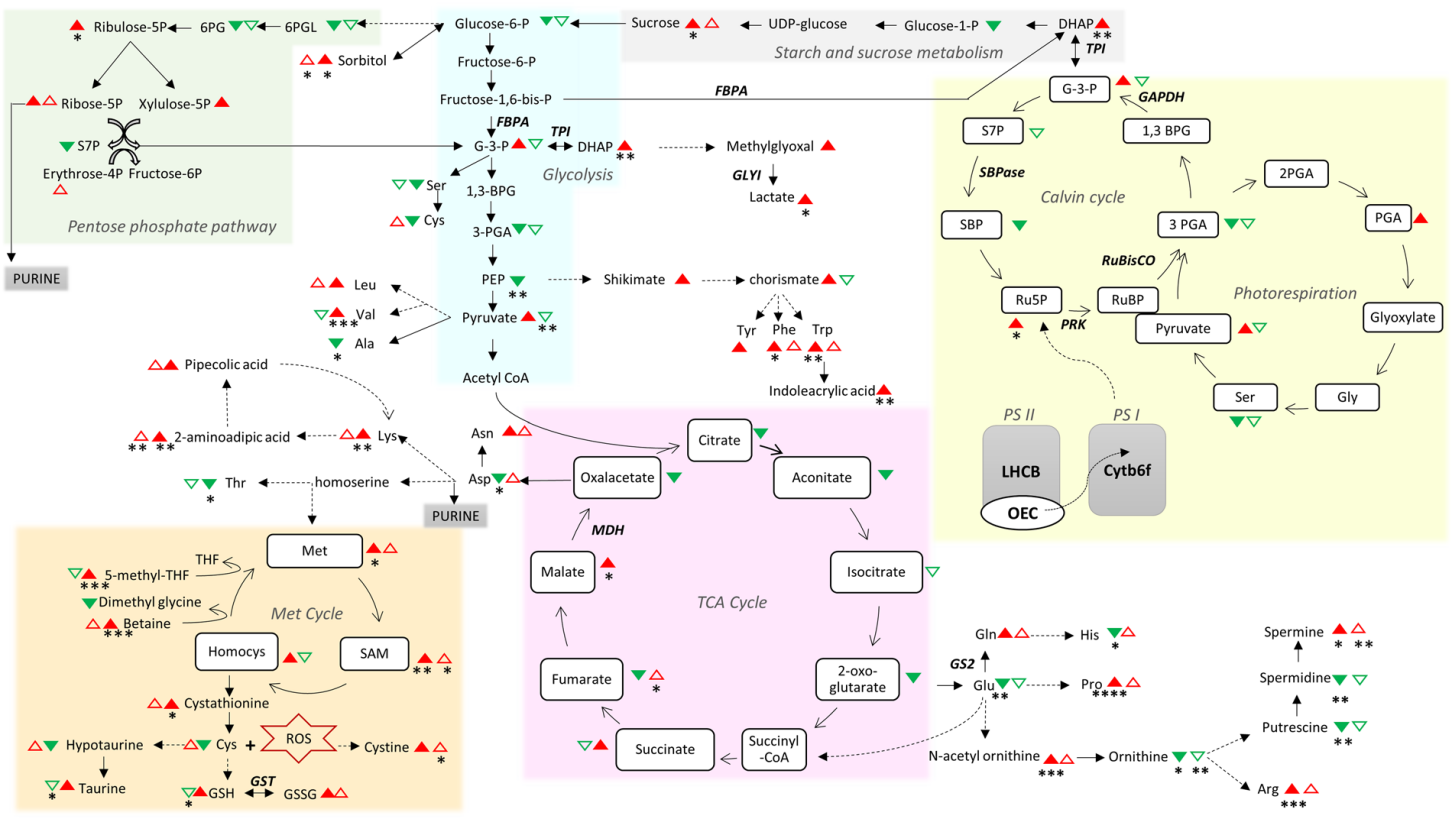
Summary scheme showing the main drought stress-induced changes detected at the protein and metabolite levels. Statistically differentially regulated proteins are displayed in italic. Color filled and outline triangles indicate Bahar and Kavir metabolite variations, respectively. Up-modulations are in red, whereas down-modulations are in green. Asterisks indicate statistically significant metabolites (*p < 0.05; **p < 0.01; ***p < 0.001; ****p < 0.0001). Solid and dashed lines indicate single- and multi-step reactions, respectively. Raw quantification data of shown metabolites are available as Supplementary Table S2.

Metabolites were extracted from leaf samples in three replicates for each of the experimental groups (including control and drought stressed Bahar plants, control and drought stressed Kavir plants) and were analyzed by LC-MS. More than 300 peaks per sample were obtained referring to the KEGG database; among them, 165 (in Bahar) and 146 (in Kavir) metabolites were analyzed more precisely and identified. In order to reduce the dimensionality of the data and visualize sample grouping, we performed an unsupervised multivariate data analysis on the LC-MS generated data and results are shown in Fig. 3 (upper panels). According to the PCA (Principal Component Analysis) models, 5 principal components (PCs) were gained from the comparison between control and water-deficit condition. In Bahar the 80.6% of variance was captured by the first two PCs, whereas the percentage was 72.8% when looking at the Kavir comparison. To confirm PCA results with a more powerful pattern recognition method, we performed a supervised PLS-DA (Partial Least Square Discriminant Analysis) and the obtained score plots are shown in Fig. 3 (lower panels). The prediction accuracies were assessed by cross validation with different numbers of components (Supplementary Fig. S3). Although the best performance could be obtained with five components, satisfactory modeling and prediction results were already gained with two PCs (accuracy 1, R2 > 0.90, Q2 > 0.81; Supplementary Fig. S3) when data were analyzed using control and drought-stressed samples for the susceptible cultivar. This indicates that metabolomes under control and water-deficit conditions are largely distinguishable in Bahar leaves. In Kavir control *vs* stressed comparison, sample groups could be separated in the PLS-DA biplot despite minor overlap. Nevertheless, the R2 and Q2 values were only 0.75 and 0.51 respectively (Supplementary Fig. S3), indicating a less metabolic perturbation under drought stress in the tolerant wheat variety with respect to the sensitive one. As a supervised method, PLS-DA also enables the selection, in the data, of the most predictive or discriminative features that are potentially useful in helping sample classification. Fundamentally, a measure of the variable importance in the PLS-DA is the VIP (variable of importance in prediction) score. On the basis of the parameter VIP > 1^30^, 16 and 14 drought-responsive metabolites with important variations were identified in Bahar and Kavir stressed leaves, respectively (Fig. 4). The changed metabolites were mainly amino acids, organic acids, and sugars. A more detailed analysis of the relevant pathways and networks affected by drought was performed by the web-based tool MetPA (Metabolic Pathway Analysis) which combines results from a powerful pathway enrichment and topology analysis. The statistical test performed was hypergeometric distribution and raw p-values < 0.05 represented significant enrichment of certain metabolites in a pathway. Moreover, since many pathways are tested at the same time, the statistical p-values from enrichment analysis were further adjusted via False Discovery Rate (FDR) estimation. Interestingly, pathway topology analysis showed that 18 and 2 canonical pathways were significantly perturbed under water-deficit conditions (FDR < 0.05; pathway impact values ≥ 0.2) in Bahar and Kavir cultivars, respectively (Table 3; Fig. 5). Main changes at the level of single metabolites are reported in Fig. 6 and discussed.

## Discussion

Drought resistance is a crucial feature in inhospitable habitats, such as dry regions of the world. In these areas, programmed selection is made for the improvement of crop drought tolerance, through precise strategies of stress-testing. In Iran, drought stress represents a relevant agronomic problem, being 60% of the total wheat area under rainfed cultivation. Quite recently, the troubleshootings related to dry farming have found their institutionalization in several organizations, whose major aim is to develop cereal cultivars perfectly fitting in every microenvironment. The introduction of improved wheat varieties has led to a significant yield increase (from 10 to 30% with respect to the local cultivars), and even more relevant if associated with improved agronomic practices such as weed control and soil fertilization. In our study we used two Iranian wheat cultivars (*i*.*e*. Kavir and Bahar), whose planting is recommended in warm-winter areas of the country, but with different rainfall levels. In fact, if Kavir has high yield and superior grain quality in regions experiencing water scarcity and salinity, Bahar performs well in irrigation conditions and is somewhat tolerant to terminal (end-of-season) drought. Among more than 20 tested spring-wheat lines, Kavir and Bahar were previously selected as the most and least drought-tolerant genotypes, respectively^29,31,32^. However, our work was carried out at the seedling stage and at more severe stress conditions than those adopted in previous investigations^33^. Moreover, it was the first time that combined proteomics and metabolomics were applied to better highlight the resistance differences between these two wheat varieties. Generally, measurements of morpho-physiological traits confirmed that the drought-tolerant cultivar Kavir has a superior performance under stress, and this evidence was strongly supported by results obtained by using omics technologies.

### Proteomic responses of wheat leaves to drought stress

It is well known that inhibition of photosynthesis is one of the primary detrimental effects of water deficit stress due to stomatal closure^34,35^ that represents the earliest response to drought causing gas exchange limitations. CO_2_ uptake by leaves and intracellular CO_2_ concentration are consequently diminished^36^. Accordingly, a general decrease trend of photosynthetic-related proteins was found by proteomics in our study as a consequence of water stress. This finding was typical of both the examined cultivars, although it appeared more exacerbated in the sensitive one (Bahar seedlings). This reduction in abundance mainly involved ribulose-1,5-biphosphate carboxylase/oxygenase (RuBisCO; at the level of intact protein and its fragments), along with other carbon fixation enzymes (fructose-1,6-bisphosphate aldolase, glyceraldehyde-3-phosphate dehydrogenase, sedoheptulose-1,7-bisphosphatase, phosphoribulokinase). Interestingly, as previously observed in barley^37^, the drought-susceptible wheat cultivar (Bahar) also showed a down-modulation of RuBisCO activase, the ATP-dependent protein responsible for the release of inhibitory sugar phosphates from the catalytic site of RuBisCO, leading to RuBisCO activation by CO_2_ (via carbamylation)^38^. Thus RuBisCO activase decrease seems strictly connected to the strong inhibition of photosynthesis registered in drought-stressed leaves. Biochemical limitations of photosynthesis due to drought stress also include photoinhibition^3,4^, damage of photosystems and decrease in the capacity to generate ATP and reductants^34,39,40^. These conditions were confirmed in our study by the detection of a down-regulation of light-harvesting complexes, of proteins essential for photosystem-II assembly and stability (*i*.*e*. CYP38 and HCF136) and of some ATP synthase subunits, respectively (see Tables 1 and 2). The reduction in abundance of these polypeptides has previously been reported in various species under drought stress^10,41–48^, however of particular note is the impairment of the ATP generation that seems to be particularly present in the sensitive cultivar. In fact, Bahar stressed leaves showed not only a marked decrease in the ATP synthase beta subunit (spots 395,396,397,402; Table 1), which is composed by the catalytic and ADP-binding unit for the conversion of ADP to ATP, but also a specific down-regulation of the ribulose biphosphate (RuBP)-producing enzyme phosphoribulokinase (spots 552, 557 and 558, Table 1). This possibly demonstrates that the photosynthetic assimilation of CO_2_ by drought stressed leaves is not so much limited by restricted CO_2_ diffusion, but rather by inhibition of RuBP synthesis, related to lower ATP content resulting from loss of ATP synthase complex^44^. Obviously, strictly associated to photosynthesis impairment, there are the biochemical changes registered at the level of nitrogen and sugar metabolism. Interestingly, drought stress led to a severe decrease in the enzyme glutamine synthetase (GS), especially in the susceptible wheat cultivar (Bahar). Recently, GS has been designated as a good metabolic indicator of drought stress tolerance in wheat; in particular, during water deficit, drought-sensitive wheat varieties showed a considerable decline of both the abundance and activity of the plastidic isoform GS2^49^, in the youngest leaves. Accordingly, significant decreases in the protein abundance of GS have been reported by proteomics in many plant species^4,50^. As a whole, under water deficit the balance between photosynthetic carbon uptake and the use of photoassimilates by the sinks is affected, causing alterations in the sugar pools in various plant compartments. Starch synthesis is generally repressed and the levels of sucrose are almost completely depleted during drought stress in a number of plant species^51,52^. However, there are also indications that in early stages of water stress a transitory increase in starch concentrations may occur^40^. In the sensitive wheat variety (Bahar), we found a decrease in abundance of the ADP-glucose pyrophosphatase (AGPPase; spot 1154, Table 1), the enzyme that catalyzes the hydrolytic breakdown of ADP-glucose (ADPG). AGPPase competes with starch synthase for ADPG, thus markedly blocking the starch biosynthesis^53^. On the other hand, AGPPase acts at a branchpoint because the final products of its catalysis are glucose-1-phosphate and AMP, two metabolic intermediates that can be diverted into numerous metabolic pathways in response to biochemical needs^52^. Interestingly, both tolerant and sensitive cultivar showed a down-regulation of the soluble inorganic pyrophosphatase during drought stress, a feature linked to several metabolic perturbations, including decreased starch content, alterations in chlorophyll and carotenoid biosynthesis, impairment in carbon assimilation ad RuBisCO translation^54^. In turn, drought-stress-induced inhibition of CO_2_ assimilation coupled with changes in photosystem activities and photosynthetic transport capacity result in an increased production of free radicals via the chloroplast Mehler reaction^55^. The ability to efficiently scavenge high levels of intracellular ROS relies on the enhanced expression of antioxidant proteins, a feature characterizing stress-tolerant crop varieties. Our results highlighted a down-regulation of the glutathione S-transferase (GST) and glyoxalase enzymes in stressed Bahar leaves, indicating the difficulty of the sensitive cultivar in detoxifying toxic molecules. GST catalyzes reactions between glutathione and a number of xenobiotics, playing a crucial role in the degradation of hazardous substances, so its down-regulation only in drought-stressed Bahar leaves is not surprising and in line with previous evidence that overexpression of the GST gene improved drought tolerance in tobacco^12^ and Arabidopsis^56^ species. Among glutathione-dependent responses of plants to drought stress there is the methylglyoxal-scavenging detoxification system^57^ that comprises glyoxalase I (GLYI) and glyoxalase II (GLYII) enzymes and has evolved to convert toxic methylglyoxal into D-lactate^58^. Over-expression of glyoxalase genes in plants has been shown to confer tolerance to multiple stresses by resisting an increase in methylglyoxal levels and maintaining redox homeostasis^59^. Our work revealed a down-regulation of GLYI only in stressed wheat leaves of the drought-sensitive cultivar Bahar (spot 667, Table 1). This finding acquires particular relevance if considered together with the decrease of the glycolytic enzyme triose phosphate isomerase (TPI; spot 840, Tables 1, 2). In fact, since methylglyoxal is generated from the triose sugars via dissociable intermediate of the reaction catalyzed by TPI in glycolysis, a decrease in the activity of TPI, concurrently with an impairment in the glyoxalase pathway, may lead to accumulation of cytotoxins in the system along with decreasing the plant energy status^54^. The evidence of a general decline in defense mechanisms upon stress in Bahar seedlings was additionally supported by the detection of a decrease in abundance of the harpin binding protein-1 (spot 770, Table 1) that is known to trigger the hypersensitive response in plants^60^. Over-expression of the harpin-encoding gene *hrf1* in rice plants showed improved drought tolerance along with increased stomatal closure and ABA, proline, and soluble sugar contents^61^. Multiple independent observations on the improvement of abiotic stress tolerance by pathogenic-related genes suggested an overlapping regulatory cascade between biotic and abiotic stresses^62^.

**Table 2:** List of differentially abundant proteins identified by LC-MS/MS in Kavir cultivar.

**Spot No**.^**a**^	Accession number	Protein name	Mascot score	Match^b^	Unique Sequences^b^	Seq. Coverage	Theoretical/Experimental Mr (kDa)	Theoretical/Experimental pI	Taxonomy	Fold change^c^	One-way ANOVA (p-value)
1311	XP_020199114.1	Probable ribose-5-phosphate isomerase 3, chloroplastic isoform X1	315	6(6)	4(4)	11%	29.898/29	7.03/4.8	Aegilops tauschii subsp. tauschii	2.5	0.006
992	P07398.1	Ribulose bisphosphate carboxylase small chain clone 512	64	1(1)	1(1)	7%	13.275/19	5.84/5.6	Triticum aestivum	1.5	0.028
577	EMT11738.1	Fructose-bisphosphate aldolase, cytoplasmic isozyme 1	474	12(12)	5(5)	11%	37.227/39	6.38/6.4	Triticum tauschii	1.9	0.030
575	EMT17623.1	Sedoheptulose-1,7-bisphosphatase, chloroplastic	218	4(4)	3(3)	6%	65.033/39	5.36/5.35	Triticum tauschii	1.6	0.033
1151	XP_020187838.1	Fructose-1,6-bisphosphatase, chloroplastic	140	5(5)	2(2)	8%	44.785/64	5.10/4.9	Aegilops tauschii subsp. tauschii	1.8	0.043
672	XP_020156816.1	Remorin-like	60	1(1)	1(1)	3%	23.386/34	5.36/5.45	Triticum tauschii	1.7	0.053
876	EMS56059.1	Chloroplast chlorophyll a-b binding protein, partial	400	18(18)	5(5)	34%	20.709/27	6.34/5.5	Triticumurartu	1.5	0.020
1173	XP_020201330.1	Cytochrome b6-f complex iron-sulfur subunit, chloroplastic	252	6(6)	4(4)	15%	24.110/22	8.47/6.25	Triticum aestivum	3.1	0.029
1313	EMS59167.1	Peptidyl-prolyl cis-trans isomerase CYP38, chloroplastic	347	8(8)	5(5)	1%	46.100/40	4.82/4.7	Triticum urartu	4.6	0.034
1227	XP_020163505.1	Photosystem II stability/assembly factor HCF136, chloroplastic	927	31(31)	11(11)	32%	42.03/40	6.47/5.3	Triticum tauschii	1.9	0.040
604	P13564.2	Full = Glutamine synthetase leaf isozyme, chloroplastic	431	12(12)	6(6)	19%	47.406/38	5.11/4.9	Hordeum vulgare	1.6	0.009
605	P13564.2	Full = Glutamine synthetase leaf isozyme, chloroplastic	204	5(5)	3(3)	11%	47.406/38	5.11/4.9	Hordeum vulgare	2.0	0.053
402	CAA52636.1	ATP synthase beta subunit	844	27(27)	12(12)	31%	59.33/50	5.56/5.3	Triticum aestivum	1.6	0.038
1299	EMT33760.1	ATP synthase delta chain, chloroplastic	242	7(7)	3(3)	25%	17.718/26	4.49/4.3	Triticum tauschii	4.8	0.027
722	XP_020167925.1	Soluble inorganic pyrophosphatase 6, chloroplastic	327	6(6)	5(5)	17%	31.825/32	5.6/5	Aegilops tauschii subsp. tauschii	2.3	0.031
840	P46226.3	Full = Triosephosphate isomerase, cytosolic	79	2(2)	2(2)	10%	27.138/28	5.24/5.8	Secale cereale	1.6	0.022
1334	ACO71288.1	cp31BHv, partial	154	4(4)	2(2)	14%	18.993/30	4.85/4.55	Triticum aestivum	2.8	0.048

^a^Spot numbers refer to Supplementary Figure S1; ^b^Values between parentheses indicate statistically significant peptides (p < 0.05); ^c^Fold of protein variation refers to stressed *vs* control. All spots resulted to be down-modulated.

**Table 3:** Detailed results from the Metabolomic Pathway Analysis (MetPA).

Cultivar	Pathway	Total Cmpd	Hits	Raw p-value	−log(p)	FDR	Impact value
Bahar	Arginine and proline metabolism	37	12	8.22E − 05	9.41E + 00	4.61E − 03	0.46
	Glycolysis or Gluconeogenesis	25	6	2.92E − 04	8.14E + 00	8.17E − 03	0.15
	Glycine serine and threonine metabolism	29	9	4.88E − 04	7.62E + 00	9.11E − 03	0.47
	Aminoacyl-tRNA biosynthesis	67	19	1.23E − 03	6.70E + 00	1.14E − 02	0.42
	Pantothenate and CoA biosynthesis	16	4	1.25E − 03	6.68E + 00	1.14E − 02	0.17
	Fructose and mannose metabolism	18	4	1.34E − 03	6.61E + 00	1.14E − 02	0.22
	Glutathione metabolism	26	8	1.60E − 03	6.44E + 00	1.14E − 02	0.41
	Alanine aspartate and glutamate metabolism	21	11	1.64E − 03	6.41E + 00	1.14E − 02	0.70
	Butanoate metabolism	20	3	1.83E − 03	6.31E + 00	1.14E − 02	0.12
	Purine metabolism	55	15	2.44E − 03	6.02E + 00	1.36E − 02	0.30
	Valine leucine and isoleucine biosynthesis	26	5	3.43E − 03	5.68E + 00	1.66E − 02	0.25
	Tryptophan metabolism	25	2	3.65E − 03	5.61E + 00	1.66E − 02	0.25
	Porphyrin and chlorophyll metabolism	33	1	3.85E − 03	5.56E + 00	1.66E − 02	0.04
	Glucosinolate biosynthesis	8	1	4.34E − 03	5.44E + 00	1.73E − 02	0.25
	Valine leucine and isoleucine degradation	34	2	4.81E − 03	5.34E + 00	1.80E − 02	0.05
	Phenylalanine tyrosine and tryptophan biosynthesis	22	9	6.13E − 03	5.09E + 00	2.15E − 02	0.38
	Galactose metabolism	26	4	7.80E − 03	4.85E + 00	2.57E − 02	0.14
	Starch and sucrose metabolism	25	2	9.34E − 03	4.67E + 00	2.91E − 02	0.07
	Lysine biosynthesis	9	2	1.06E − 02	4.55E + 00	3.11E − 02	0.12
	Carbon fixation in photosynthetic organisms	21	8	1.19E − 02	4.43E + 00	3.34E − 02	0.34
	Thiamine metabolism	10	2	1.34E − 02	4.31E + 00	3.52E − 02	0.33
	Nitrogen metabolism	16	4	1.58E − 02	4.15E + 00	3.52E − 02	0.20
	Vitamin B6 metabolism	11	1	1.58E − 02	4.15E + 00	3.52E − 02	0.14
	Selenoamino acid metabolism	18	1	1.61E − 02	4.13E + 00	3.52E − 02	0.00
	Glycerophospholipid metabolism	25	3	1.62E − 02	4.12E + 00	3.52E − 02	0.10
	Cysteine and methionine metabolism	35	10	1.63E − 02	4.12E + 00	3.52E − 02	0.42
	Cyanoamino acid metabolism	11	3	1.89E − 02	3.97E + 00	3.92E − 02	0.17
	Histidine metabolism	16	4	2.49E − 02	3.69E + 00	4.45E − 02	0.23
	Nicotinate and nicotinamide metabolism	10	2	2.55E − 02	3.67E + 00	4.45E − 02	0.25
	Phenylpropanoid biosynthesis	31	2	2.56E − 02	3.67E + 00	4.45E − 02	0.06
	Phenylalanine metabolism	11	2	2.58E − 02	3.66E + 00	4.45E − 02	0.33
	Terpenoid backbone biosynthesis	24	2	2.61E − 02	3.65E + 00	4.45E − 02	0.08
	Pentose phosphate pathway	17	3	2.62E − 02	3.64E + 00	4.45E − 02	0.21
Kavir	Tyrosine metabolism	18	3	7.63E − 04	7.18E + 00	3.81E − 02	0.08
	Zeatin biosynthesis	16	1	1.88E − 03	6.28E + 00	3.81E − 02	0.00
	Purine metabolism	55	18	2.16E − 03	6.14E + 00	3.81E − 02	0.30
	C5-Branched dibasic acid metabolism	4	1	3.96E − 03	5.53E + 00	4.19E − 02	0.50
	Glycolysis or Gluconeogenesis	25	1	3.96E − 03	5.53E + 00	4.19E − 02	0.03
	Ubiquinone and other terpenoid-quinone biosynthesis	22	2	4.82E − 03	5.33E + 00	4.26E − 02	0.11

Only pathways with FDR < 0.05 are shown.

### Leaf metabolome changes induced by drought stress

Considering that MetPA functionally grouped only the pyruvic acid as unique hit into the KEGG pathway referred to C5-branched dibasic acid metabolism in the Kavir control *vs* stress comparison, we can affirm that purine metabolism was the pathway basically changed by drought stress in the drought-tolerant cultivar. The results from the current investigation showed that upon drought stress, major purine bases (adenine and guanine) are up-regulated compared to the control, clearly indicating the activation of a tolerance mechanism to protect nucleic acids, as recently observed in soybean^63^. However, this trait was not exclusive to this variety since purine metabolism was detected as significantly altered also in Bahar stressed leaves. Very interestingly, 9 of the top metabolic pathways of importance in the drought-sensitive cultivar (Bahar) were related to amino acid metabolism, including: (i) arginine and proline metabolism; (ii) alanine, aspartate and glutamate metabolism; (iii) glycine, serine and threonine metabolism; (iv) cysteine and methionine metabolism; (v) phenylalanine, tyrosine and tryptophan biosynthesis. Levels of several amino acids significantly increased during drought stress, especially in Bahar leaves (Fig. 6; Supplementary Table S2). This can reasonably result from enhanced stress-induced protein breakdown. However, pool size of free amino acids can also depend on extensive regulation of biosynthetic processes, as well documented in the case of connected proline and arginine metabolisms^27,64,65^. One of the well-known biomarkers for water deficit due to its osmoprotectant role is exactly proline, that dramatically increases in Bahar (p < 0.0001; 3-fold) and Kavir (ns; 2-fold) stressed leaves when compared to their corresponding controls and is second and fourth in variable importance of prediction, respectively (Fig. 4). As a compatible solute, proline basically protects cellular structures during dehydration and it is essential for osmotic adjustments^66^, however, it also results to be a potent ROS scavenger (in particular towards singlet oxygen), providing evidence that it is an important contributor to cellular redox balance under stress conditions^67^. Oxidative stress perturbs the TCA cycle^65,68^ and indeed TCA-cycle-derived amino acids aspartate and glutamate decreased during water-deficit conditions in Bahar seedlings (Fig. 6; Supplementary Table S2). The observed glutamate decline can be connected to alanine reduction, but also to proline and arginine accumulation registered after 7-days of water withholding (Fig. 6; Supplementary Table S2). In fact, if on one hand Glu and Ala can supply amino groups for the photorespiratory metabolism (with consequent serine-derived pyruvate increase)^65,69,70^, on the other hand the metabolism of Glu into ornithine to produce Pro and/or Arg constitutes one of the major interactive pathways for carbon (C) and nitrogen (N) assimilation and partitioning. This group of sub-pathways plays critical roles not only in plant development, but also in plant stress response^71^ because it represents the primary source of putrescine (Put) biosynthesis, which in turn produces the other two common polyamines (PAs), spermidine (Spd) and spermine (Spm). These molecules, along with Pro, glycine betaine, and some sugars (*e*.*g*.sorbitol, trehalose, raffinose), are considered key osmoprotective elements and their concentrations are known to markedly increase in several plant species under drought stress conditions^72^, as well as in our investigation (Fig. 6). According to this route, we found an up-regulation of the intermediate N-acetyl-ornithine (which is also one of the most important features associated with drought in both Bahar and Kavir leaves with a VIP of 1.24 and 1.42, respectively; Fig. 4), and a down-regulation of ornithine (precursor of Put-Spd-Spm; Fig. 6). On the other hand, the decrease in aspartate amounts opens another interesting clue that is the accumulation of lysine but also the activation of the saccharopine pathway in Bahar drought-stressed leaves. In fact, cereals synthesize lysine from aspartate, but lysine is catabolized through the saccharopine pathway^73^ into aminoadipic acid (Fig. 6). Levels of aminoadipic acid strongly increased in both Bahar (2.5-fold) and Kavir (4-fold) drought stressed leaves (Supplementary Table S2) and this agrees with previous observations demonstrating an induction of the α-aminoadipic-δ-semialdehyde dehydrogenase (AASADH, the last enzyme of the saccharopine pathway directly responsible for aminoadipic formation) when plants are submitted to osmotic, salt, and drought stresses^74^. Also, osmotic shock was found to induce the activity of the bifunctional enzyme LKR/SDH^75^, by likely channeling Lys to the formation of pipecolic acid which acts as osmoprotectant^76^ (Fig. 6). In Bahar stressed leaves we observed an accumulation of all the aromatic amino acids (Trp, Phe, Tyr). Aromatic amino acids are synthesized in plants through the shikimate pathway. In support of an up-regulation of shikimate pathway under withholding water conditions, Bahar metabolome analysis revealed a marked increase of the shikimate and chorismate intermediates (Fig. 6; Supplementary Table S2). Aromatic amino acids are target of oxidation, and in free-form they may have a protective function against ROS. This role of buffer between ROS and proteins is especially played by tryptophan in the chloroplast^77^. Trp is one of the top important features associated with drought stress both in Bahar and Kavir analyses (Fig. 4). According to our results, dramatic increases of Trp concentration under water-deficit conditions were previously found in wheat and maize cultivars of differing drought tolerance^25,78^, spotting few adverse effects on plant growth. Aromatic amino acids also serve as precursors of a wide range of secondary metabolites such as auxins, terpenoids, glycosides and lignin building blocks^79^. Among metabolites with the highest VIP scores in Bahar control *vs* drought stress analysis, we found the indoleacrylic acid (Fig. 4), a believed naturally occurring auxin generated from Trp via a two-step pathway^80^. Pathway analysis finally highlighted the up-regulation of another route that it is worthwhile to mention, *i*.*e*. the methyl cycle. Specifically, we detected an increase of methionine, cystathionine, S-adenosyl-L-methionine (SAM), but especially 5-methyl-tetrahydrofolate (THF; Fig. 6). Although there are few and ambiguous data on free Met accumulation under drought^81^ the enzyme methionine synthase was found to increase in leaves under conditions of water deficit^10,11^. Of particular relevance is the accumulation of SAM for its possible role in feeding polyamines production^82^ to cope with drought stress.

In conclusion, our work is a contribution to the ongoing efforts elucidating the biochemical complex mechanisms underlying plant responses to drought stress by exploiting an integrated multiple-omics analysis. Based on our results, suggested strategies for engineering wheat tolerance to water stress are the: (i) maintenance of RuBP synthesis; (ii) overexpression of AGPPase for starch biosynthesis control; (iii) functionality increase of glutathione-dependent responses; (iv) accumulation of organic osmolytes and (v) down-regulation of auxin (indoleacrylic acid) production. This study provided information on several metabolites which can be useful for the development of ameliorated models establishing the connection between yield-associated traits and various metabolic pathways. Surely, we still are at the beginning of using omics-assisted breeding to obtain stress-resistant cultivars, but its role in crop improvement will become increasingly evident in the future.

## Materials and Methods

### Plant material and morpho-physiological trait measurements

For germination, seeds were distributed in a 10-cm-diameter sterile Petri dish with two layers of saturated filter paper. The petri dish was placed in an incubator at 25 °C ± 1 with 45% relative humidity and the surface of seeds was wet with 5cc of water every day, for one week. Then, seeds with the same bud were transferred to loam soil containing P_2_O_5_ 160 mg/kg, K_2_O 180 mg/kg, KCl 0.9 g/kg, and CaCl_2_ 140 mg/kg. Each spring-wheat variety was cultivated in a split plot basis of randomized complete block design with three replications, under two different conditions (normal irrigation and water deficit). Ten germinated seeds were planted in each experimental unit. Plants were grown in an experimental greenhouse under 40% humidity, in 16-hour daylight at 25 °C, and with light intensity of 300 µmol m-2 s-1. Irrigation daily testing was performed for all the units. After ten days of planting, drought was imposed by withholding water for a week. During this period the control units continued irrigation. Before sampling, the crown height was measured to the highest leaf plantlets. Plant fresh weight (PFW), dry weight (PDW), and leaf relative water content (RWC) were measured according to Morant- Manceau *et al*.^83^. Leaf temperature measurement was made using an infrared thermometer. The chlorophyll index was determined using a chlorophyll meter (SPAD-502, Japan). Osmotic potential was measured by osmometer (model: Osmomat 010, Gonotec) according to Martinez *et al*.^84^. Measurements of specific leaf area (SLA) trait was performed by calculating the ratio of leaf area to leaf dry weight (cm^2^ g^−1^). A two-way ANOVA analysis was performed by using the GraphPad software (version 5). Duncan’s multiple comparison post-hoc test was performed with the DSAASTAT macro (version 1.1) developed by Onofri^85^.

### Protein extraction and solubilization

1 g of young wheat leaves randomly collected from plants in a single pot was finely ground in liquid nitrogen and the protein extraction was performed as exactly reported by Rinalducci *et al*.^86^. Three biological replicates (different pots grown side by side in the same growth chamber) were used. The wheat leaf proteins in the dried powder were solubilized in 9 M urea, 4% CHAPS, 1% DTT, 1% pH 3–10 ampholytes (Bio-lyte; Bio-Rad), 35 mM Tris base via incubation at 37 °C for 1 h with continuous stirring. The mixture was centrifuged at 12 000 × g at room temperature for 15 min and a small aliquot was used to determine the protein content by the Bradford assay^87^.

### 2D gel electrophoresis and image analysis

IEF was performed using Bio-Rad Multiphore II and Dry Strip Kit (Bio-Rad-Protean-IEF-Cell-System). Seventeen centimeters IPG strips (Bio-Rad, Hercules, CA, USA) pH 4–7 were passively rehydrated overnight with 600 μg of protein in 300 μl of solubilisation solution containing 1% carrier ampholyte (Bio-lyte 4–7; Bio-Rad, Hercules, CA, USA), 2 M Urea, 7 M Thiourea and 4% CHAPS. The total product time × voltage applied was 80 000 V h for each strip at 20 °C. Strips were subsequently reduced (1% DTT, 15 min) and alkylated (2.5% IAA, 15 min) during the equilibration step (30 min in 50 mM Tris-HCl pH 8.8, 6 M urea, 30% glycerol v/v, 1% SDS, bromophenol blue). Equilibrated strips were then placed on SDS-polyacrylamide gels, 18.5 cm × 20 cm, 13% acrylamide, and sealed with 0.5% agarose. SDS-PAGE was performed using the Bio-Rad Protean II XL Cell, large gel format, at constant current (40 mA per gel) at 7 °C until the bromophenol blue tracking dye was approximately 2–3 mm from the bottom of the gel. Protein spots were stained with colloidal Coomassie Brilliant Blue G-250. To ensure protein pattern reproducibility, three technical replicates were done. The scanned gel images were transferred to the Progenesis SameSpots software package (Nonlinear Dynamics, Newcastle, UK), which allows spot detection, background subtraction, and protein spot OD intensity quantification (spot quantity definition). The gel image showing the highest number of spots and the best protein pattern was chosen as a reference template and the images were aligned onto it. Spot quantity values were normalised in each gel dividing the raw quantity of each spot by the total quantity of all the spots included in the standard gel. For each protein spot, the average spot quantity value and its variance coefficient in each group was determined. One-way analysis of variance (ANOVA) was carried out at p < 0.05 in order to assess for absolute protein changes among control versus drought-stressed samples; only 1.5-fold or higher quantitative variations were taken into consideration.

### In-gel digestion and LC-MS/MS analysis

Gel bands were carefully excised from the gel and subjected to in-gel trypsin digestion according to Shevchenko *et al*.^88^. Peptide extracts were analyzed by using a split-free nano-flow liquid chromatography system (EASY-nLC II, Proxeon, Odense, Denmark) coupled with a 3D-ion trap (model AmaZon ETD, Bruker Daltonik, Germany) equipped with an online ESI nanosprayer (the spray capillary was a fused silica capillary, 0.090 mm OD, 0.020 mm ID) in the positive-ion mode. For all experiments, a sample volume of 15 μl was loaded by the autosampler onto a homemade 2-cm fused silica precolumn (100 μm I.D.; 375 μm O.D.; Reprosil C18-AQ, 5 μm, Dr. Maisch GmbH, Ammerbuch-Entringen, Germany). Sequential elution of peptides was accomplished by using a flow rate of 300 nl/min and a linear gradient from Solution A (100% water; 0.1% formic acid) to 50% of Solution B (100% acetonitrile; 0.1% formic acid) in 40 min over the precolumn on-line with a homemade 15-cm resolving column (75 μm ID; 375 μm OD; Reprosil C18-AQ, 3 μm, Dr. Maisch GmbH, Ammerbuch-Entringen, Germany). The acquisition parameters for the mass spectrometer were as previously reported^86^. Acquired MS/MS spectra were processed in DataAnalysis 4.0 and submitted to the Mascot search program (Matrix Science, London, UK). The following parameters were adopted for database searches: NCBInr database (release date April 07, 2017; 5011440 sequences); taxonomy = Viridiplantae; peptide and fragment mass tolerance =  ±0.3 Da; missed cleavages = 1; fixed modifications = carbamidomethyl (C); variable modifications: oxidation (M) and significance threshold level (P < 0.05) for Mascot scores (−10 Log (P)). In the case of hits with only one statistically significant unique peptide, even though high Mascot scores were obtained with significant values, a combination of automated database searches and manual interpretation of peptide fragmentation spectra was used to validate protein assignments.

### Functional annotation

Differentially abundant protein lists were analyzed by the Gene Ontology (GO) based online tool agriGO (http://systemsbiology.cau.edu.cn/agriGOv2/)^89^ using the Singular Enrichment Analysis (SEA) category, with the following parameters: (i) Reference background: Entry identifier (Uniprot 2016); (ii) Statistical test method: Fisher; (iii) Multi-test adjustment method: Hochberg (FDR); (iv) Significance level: 0.05; (v) Minimum number of mapping entries: 5; and (vi) Gene ontology type: Plant GO Slim. Next, in order to remove the redundant GO terms, the online tool REVIGO^90^ was used with the following parameters: (i) Allowed similarity: medium (0.7); (ii) Semantic similarity measure: SimRel. As protein identifications were performed by Mascot-based homology searches, IDs from different plant species were obtained (see Tables 1–2). Thus, in order to use the most complete GO profile as background database list in the agriGO tool, BLAST runs against *Oryza sativa japonica* were performed and this species was selected for both SEA and REVIGO analysis.

### Metabolite extraction and LC-MS analysis

200 mg of leaves per condition (the same plants from which samples for proteomics analyses were collected) were finely ground in liquid nitrogen and powder was used for metabolite extractions as previously reported^91^. Briefly, the vegetable cells were lysed by thermal shock (freezing/heating). A cold (−20 °C) solution of 60% methanol/40% chloroform was added to each tube. The tubes were mixed for 30 min and subsequently centrifuged at 1000 × g for 1 min at 4 °C, before being transferred to −20 °C for 2–8 h. After thawing, liquid phases were recovered and an equivalent volume of acetonitrile was added to precipitate any residual protein. Samples were incubated at 4 °C for 20 min, centrifuged at 10,000 × g for 10 min at 4 °C and the collected supernatants were dried to obtain visible pellets. Finally, the dried samples were re-suspended in water, 5% formic acid and transferred to glass autosampler vials for LC/MS analysis. Twenty microliters of samples (3 biological replicates × 3 technical replicates × 2 conditions × 2 cultivars) were injected into an Ultra High-Performance Liquid Cromatography (UHPLC) system (Ultimate 3000, Thermo) and run in positive ion mode. A Reprosil C18 column (2.0 mm × 150 mm, 2.5 μm - Dr Maisch, Germany) was used for metabolite separation. Chromatographic separations were achieved at a column temperature of 30 °C and flow rate of 0.2 mL/min. A 0–100% linear gradient of solvent A (ddH2O, 0.1% formic acid) to B (acetonitrile, 0.1% formic acid) was employed over 20 min, returning to 100% A in 2 min and a 6-min post-time solvent A hold. The UHPLC system was coupled online with a mass spectrometer Q-Exactive (Thermo) scanning in full MS mode (2 μscans) at 70,000 resolution in the 67 to 1000 m/z range, target of 1 × 10^6^ ions and a maximum ion injection time (IT) of 35 ms. Source ionization parameters were: spray voltage, 3.8 kV; capillary temperature, 300 °C; sheath gas, 40; auxiliary gas, 25; S-Lens level, 45. Calibration was performed before each analysis against positive ion mode calibration mixes (Piercenet, Thermo Fisher, Rockford, IL) to ensure sub ppm error of the intact mass.

### Metabolomic data processing and statistical analysis

Raw files of replicates were exported and converted into mzXML format through MassMatrix (Cleveland, OH), then processed by MAVEN software (http://maven.princeton.edu/)^92^. Mass spectrometry chromatograms were elaborated for peak alignment, and tentative metabolite identification (within a 2 ppm mass-deviation range between observed and expected results against the imported KEGG database). Univariate (two-sample t-test) and multivariate (PCA, PLS-DA) statistical analyses were performed on the entire metabolomics data set using the MetaboAnalyst 3.0 software (http://metpa.metabolomics.ca/). To increase the importance of low-abundance ions without significant amplification of noise, raw data were normalized by pareto scaling. False discovery rate (FDR) was used for controlling multiple testing. Pathway analysis was performed utilizing the MetPA (Metabolomic Pathway Analysis) web-based tool^93^ incorporated into MetaboAnalyst platform. Data for identified metabolites detected in all samples was submitted into MetPA with annotation based on common chemical names. Verification of accepted metabolites was conducted manually using HMDB, KEGG, and PubChem DBs. Oryza sativa pathway library was used for pathway analysis.

## Electronic supplementary material

Supplementary Information

Supplementary Table S2
