# Supplementary material for: Metabolomics and proteomics reveal drought-stress responses of leaf tissues from spring-wheat
Source: Sci Rep. 2018 Apr 9;8:5710. doi: 10.1038/s41598-018-24012-y (PMC5890255; doi:10.1038/s41598-018-24012-y)
Supplement: Supplementary file 1 — Supplementary Information [file 41598_2018_24012_MOESM1_ESM.doc]

**SUPPLEMENTARY INFORMATION**

**Metabolomics and proteomics reveal drought-stress responses of leaf tissues from spring-wheat**

Anna Michaletti,^1^ Mohammad Reza Naghavi,^2^ Mahmoud Toorchi,^3^ Lello Zolla,^4^,**^*^** Sara Rinalducci^1,*^

*^1^ Department of Ecological and Biological Sciences (DEB), University of Tuscia*, *Viterbo, Italy*

*^2^ Department of Agriculture, Payame Noor University, Tehran, Iran*

*^3^ Department of Biotechnology and Plant Breeding, University of Tabriz, Tabriz, Iran*

*^4^* *Department of Science and Technology for Agriculture, Forestry, Nature and Energy (DAFNE), University of Tuscia, Viterbo, Italy*

**^*^** Corresponding authors:

Prof. Sara Rinalducci. Department of Ecological and Biological Sciences (DEB), University of Tuscia, Largo dell’Università snc, 01100 Viterbo, Italy. Email: [sara.r@unitus.it](mailto:sara.r@unitus.it). Phone: +39 0761 357101.

Prof. Lello Zolla. Department of Science and Technology for Agriculture, Forestry, Nature and Energy (DAFNE), University of Tuscia, Via San Camillo De Lellis snc, 01100 Viterbo, Italy. Email: [zolla@unitus.it](mailto:zolla@unitus.it). Phone: +39 0761 357100.

**Supplementary Table S1.** Descriptive statistics (mean, standard deviation, coefficient of variation) for the examined traits under well-watered (control) and drought-stress conditions.

| **Morpho-physiological trait** | **Bahar** | | | **Kavir** | | | **Control** | | | **Drought-stress** | | |
| --- | --- | --- | --- | --- | --- | --- | --- | --- | --- | --- | --- | --- |
|  | **mean** | **SD** | **CV (%)** | **mean** | **SD** | **CV (%)** | **mean** | **SD** | **CV (%)** | **mean** | **SD** | **CV (%)** |
| **SLA (cm^2^ g^-1^)** | 193,82 | 33,32 | 17,19 | 149,89 | 11,83 | 7,89 | 156,09 | 25,01 | 16,02 | 187,62 | 34,44 | 18,35 |
| **PH (cm)** | 14,58 | 2,91 | 19,93 | 16,70 | 1,53 | 9,14 | 17,15 | 1,43 | 8,36 | 14,14 | 2,45 | 17,33 |
| **PFW (g)** | 0,269 | 0,050 | 18,65 | 0,356 | 0,0279 | 7,84 | 0,336 | 0,047 | 14,13 | 0,289 | 0,065 | 22,48 |
| **PDW (g)** | 0,055 | 0,035 | 63,61 | 0,106 | 0,023 | 21,51 | 0,099 | 0,032 | 32,63 | 0,062 | 0,038 | 61,09 |
| **RWC (%)** | 73,65 | 6,67 | 9,06 | 81,71 | 3,26 | 3,99 | 82,15 | 2,77 | 3,38 | 73,20 | 6,20 | 8,47 |
| **Osmotic potential (MPa)** | -0,818 | 0,262 | 31,99 | -1,146 | 0,520 | 45,37 | -0,806 | 0,345 | 42,77 | -1,158 | 0,459 | 39,62 |
| **Leaf temperature (°C)** | 25,317 | 2,482 | 9,80 | 23,733 | 1,936 | 8,156 | 22,85 | 1,25 | 5,49 | 26,20 | 1,76 | 6,72 |
| **Chlorophyll index (SPAD)** | 41,55 | 2,01 | 4,84 | 43,47 | 1,76 | 4,05 | 43,70 | 1,92 | 4,40 | 41,32 | 1,50 | 3,63 |


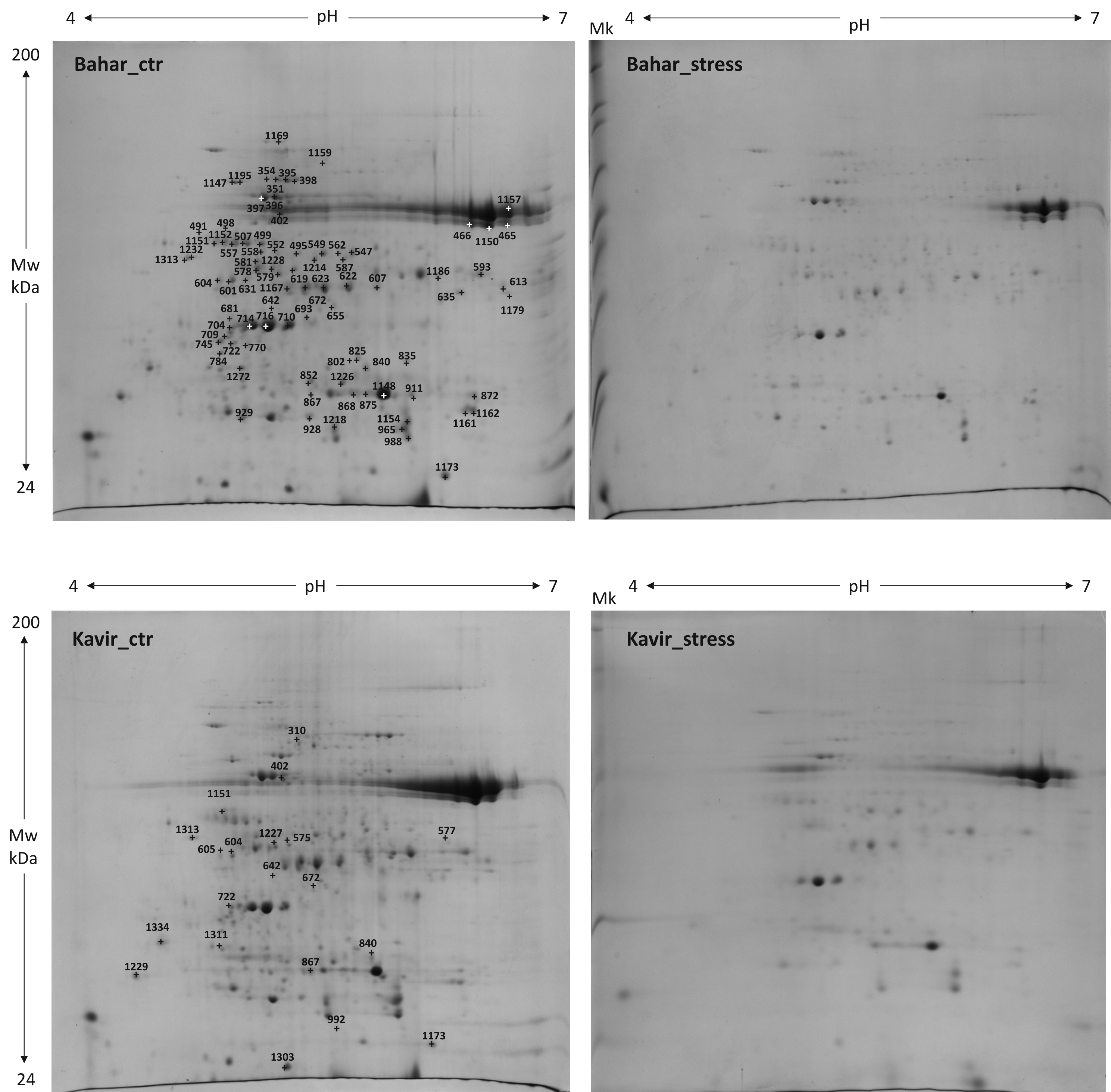


**Supplementary Figure S1. Representative 2-DE gels of control and drought-stressed leaves.** Upper panels show maps from Bahar (sensitive) cultivar, whereas lower panels display Kavir (tolerant) cultivars. Gel images were acquired by ChemiDoc XRS system (Bio-Rad Laboratories). For each wheat variety, molecular size markers were loaded onto a separated lane (Mk) only in one of the technical replicates. Numbers indicate the statistically variable protein spots as detected by using Progenesis SameSpots software. Samples used for quantitative comparisons derived from the same experiment and gels were processed in parallel.


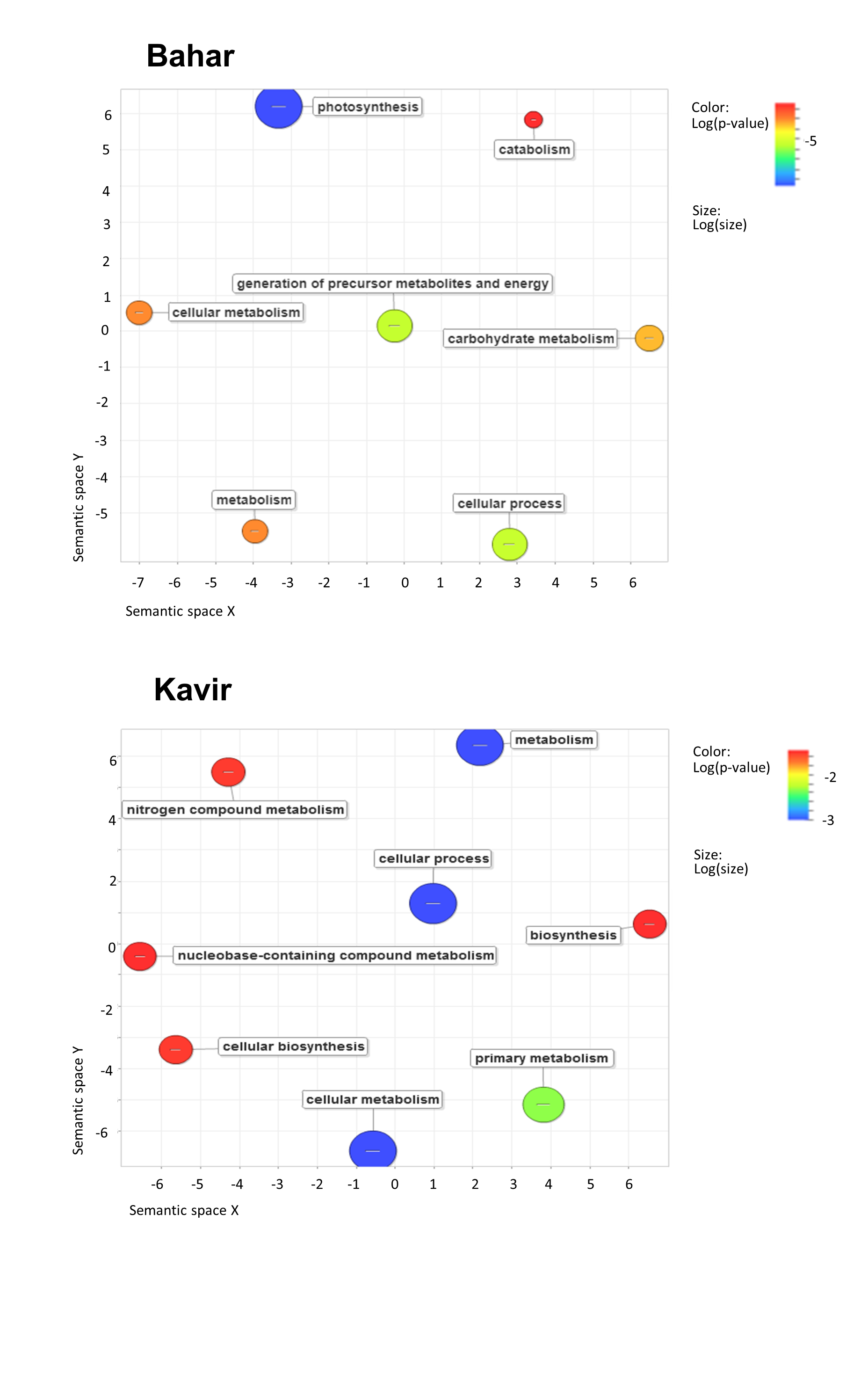


**Supplementary Figure S2. Analysis by REVIGO platform of drought stress regulated proteins.** The scatter plots show the cluster representatives (*i.e.* terms remaining after reducing redundancy) in a two-dimensional space where x and y coordinates derive by applying multi-dimensional scaling to a matrix of the GO terms semantic similarities. Bubble color indicates the Log(p-value) for the FDRs derived from the agriGO analysis. The circle size represents the frequency of the GO term in the Uniprot database, where a larger size bubble represents more general terms.


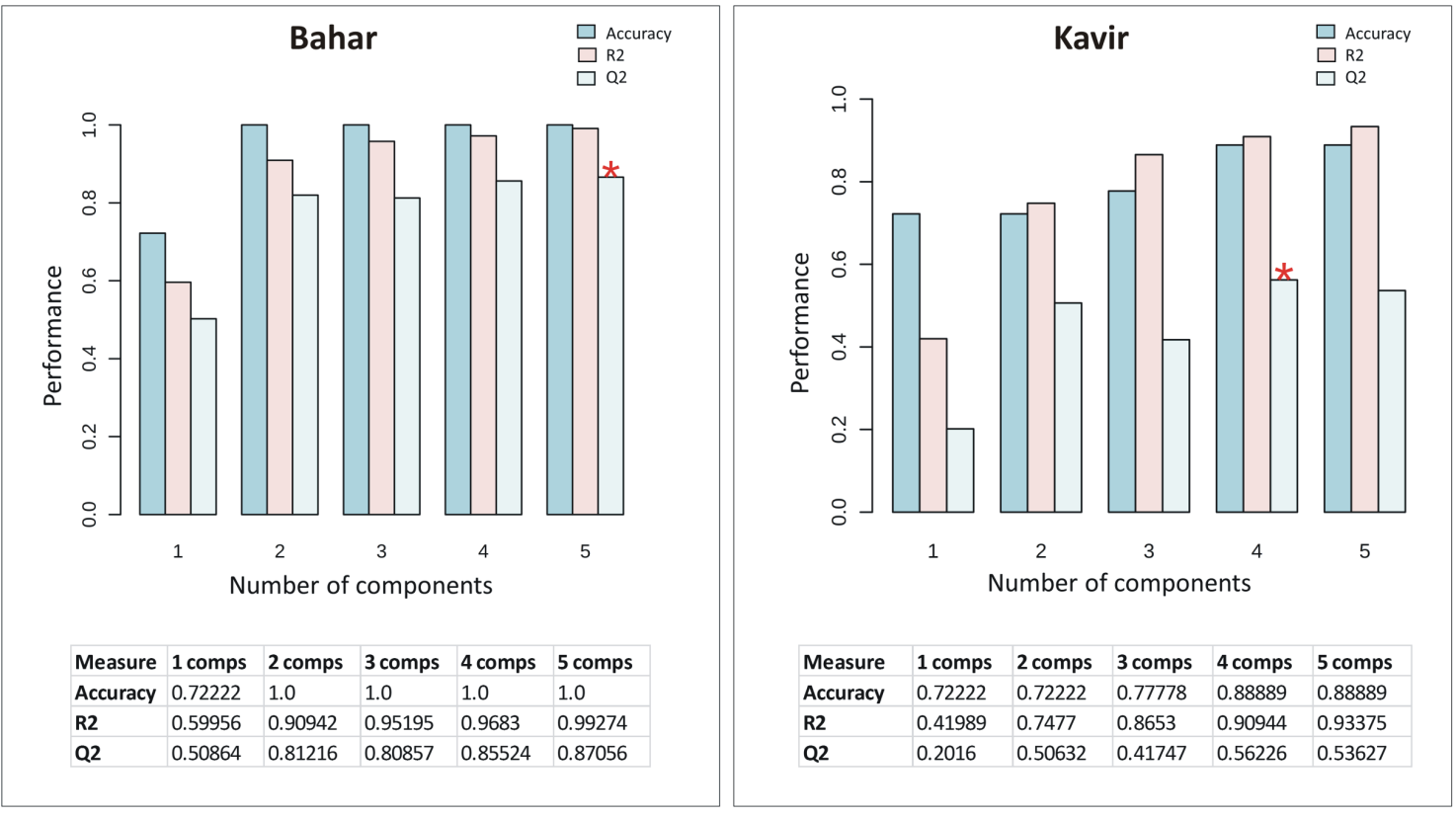


**Supplementary Figure S3.** **PLS-DA performance measurements.** Accuracy, multiple correlation coefficient R2 and the explained variance in prediction Q2 are shown. The red asterisk indicates the best value of selected measure (Q2).
